# An Analysis of Smart Contracts Security Threats Alongside Existing Solutions

**DOI:** 10.3390/e22020203

**Published:** 2020-02-11

**Authors:** Antonio López Vivar, Alberto Turégano Castedo, Ana Lucila Sandoval Orozco, Luis Javier García Villalba

**Affiliations:** 1 Group of Analysis, Security and Systems (GASS), Department of Software Engineering and Artificial Intelligence (DISIA), Faculty of Computer Science and Engineering, Office 431, Universidad Complutense de Madrid (UCM), Calle Profesor José García Santesmases, 9, Ciudad Universitaria, 28040 Madrid, Spain; alopezvivar@fdi.ucm.es (A.L.V.); albeture@ucm.es (A.T.C.); asandoval@fdi.ucm.es (A.L.S.O.); 2 Raona Enginyers S.L. Calle de Goya, 115, 2n, 28009 Madrid, Spain

**Keywords:** blockchain, Ethereum, secure development, security, smart contracts

## Abstract

Smart contracts have gained a lot of popularity in recent times as they are a very powerful tool for the development of decentralised and automatic applications in many fields without the need for intermediaries or trusted third parties. However, due to the decentralised nature of the blockchain on which they are based, a series of challenges have emerged related to vulnerabilities in their programming that, given their particularities, could have (and have already had) a very high economic impact. This article provides a holistic view of security challenges associated with smart contracts, as well as the state of the art of available public domain tools.

## 1. Introduction

Smart contracts are gaining popularity in recent times, although the original concept is relatively old. The idea of smart contracts appears in [1] and has evolved to the present day, especially after the introduction in 2009 of Bitcoin [2] and its decentralised blockchain. In short, a smart contract is a computer program that runs on a decentralised basis, modifying, if executed correctly, the overall state of the system, which is stored in a blockchain. As for the potential applications and cases of the use of smart contracts are very varied but their decentralised nature, speed, automation, absence of intermediaries, and transparency make them particularly suitable in different sectors, such as: identity management [3,4], electronic voting [5,6], banking and financial services [7], supply chain [8], IoT [9], online gaming [10,11], and medical information [12] for example. Although it is usually associated with the Ethereum platform [13], today there are many platforms that make use of them, such as Hyperledger [14] or Corda [15] among many others ([16] presents an updated list of all existing smart contract platforms). In this work, we will focus on Ethereum, although much of the security concepts can be extrapolated to other platforms.

While other academic publications tend to focus more on methods, with this article we have sought to focus on an analysis of existing tools, downloading them (those that are available), installing, and configuring them from scratch and making a brief analysis of their functionality (which will be further expanded in other work in progress by the same authors, where the real capacities of each tool will be analysed against several smart contract datasets). The remaining paper is structured as follows: Section 2 introduces distributed ledger technologies, blockchain, and smart contracts. Section 3 focuses on families of vulnerabilities, analysis methods, and the most relevant attacks that have occurred on Ethereum. In Section 4, a description and small analysis of most of the existing public domain tools for searching vulnerabilities in smart contracts are made. Finally, Section 5 presents the conclusions of this article.

## 2. Background

A quick introduction to the distributed ledger technologies, blockchain, and smart contracts is presented in this section.

### 2.1. Distributed Ledger Technology

**Figure 1:**
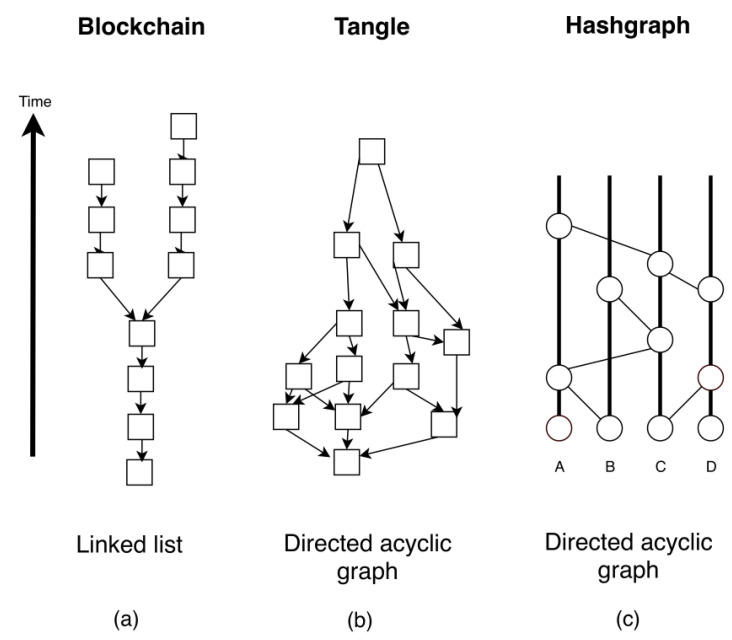
A summary of existing Distributed Ledger Technology (DLT). (**a**) Blockchain is a distributed, decentralized and immutable ledger to store trans-actions history. (**b**) Tangle is a decentralised data storage architecture and a consensus protocol, based on a Directed Acyclic Graph (DAG) data structure. (**c**) Hashgraph uses a DAG as data structure for storing transactions, and a voting algorithm combined with gossip protocol to quickly reach consensus among node [17].

A distributed ledger can be seen as a kind of data structure replicated and synchronised in multiple nodes within a network and a set of methods to manipulate them. The most notable properties of a Distributed Ledger Technology (DLT) are immutability, censorship resistance, decentralised management, and the absence of the requirement for a trustworthy third party. There are several kinds of DLT as can be seen in Figure 1 according to the type of data structure used (the most common are linked lists and directed acyclic networks) but they all share three characteristics: the usage of public key cryptography, the utilisation of P2P networks, and a consensus mechanism.

As smart contracts, and specifically Ethereum, use a blockchain, we will explain a little more in this DLT. A blockchain is basically a very big file that represents a linked list of *blocks*, replicated in multiple nodes, called *miners*, that keep a copy of it (sometimes not the full chain, just the latest n *blocks*). Operations, called transactions, are recorded in a ledger, grouped into blocks, which are added to the chain by a consensus algorithm usually based on a proof of work [18]. To perform this proof of work, each of the nodes prepare a candidate block with a set of new transactions pending to be added to the blockchain, to which it adds a counter and a pointer to the last block of the chain. Then each node executes on its candidate block a cryptographic hash algorithm (in the case of Ethereum it is KECCAK-256) [19]. If the resulting hash is less than a certain value, the block is considered mined and propagates along with the calculated hash to be verified by the rest of the nodes and to update their copy of the block string. Otherwise, if the resulting hash is not valid (most likely), the block counter is increased and the hash is recalculated. There are public, private, and hybrid blockchains depending on the type of visibility and access permissions, whose characteristics make them suitable for different applications. If it is of interest to the reader, Table 1 shows a summary of the strengths and weaknesses of blockchain and other DLTs.

### 2.2. Smart Contracts

In Ethereum, there are two types of transactions, the normal ones, where user A sends an amount of *ether* (the name given to the Ethereum cryptocurrency) to the address of user B. The user addresses basically consist of a public/private key pair and each transaction generated is digitally signed by the user sending the funds. In addition to *user addresses*, there are *contract addresses*, which, as their name indicates, point to a smart contract. To add a new smart contract to the blockchain, a user has to generate a special type of transaction with a series of data, including the source code of the compiled contract. To execute a smart contract, a user will have to create a transaction from their user address by sending *ether* (and other data required by the contract) to the contract address. If the execution of the smart contract is successful, the overall status of Ethereum will be modified. Otherwise, if the execution fails, the user will be charged the computational cost used from the *ether* that was sent in the transaction (called *gas*), but there will be no change in the overall state of the system. The concept of *gas* should be discussed. Solidity is a Turing complete language, so there is the possibility of provoking infinite loops in the execution. One way to avoid this (which could be used as a form of denial of service attack against the Ethereum network) is a mechanism whereby each bytecode operation has a pre-set cost called *gas*. When a user invokes a contract, he/she has to pass the unit price of the *gas* and the *gas* limit she/he is willing to assume. If during contract execution, the *gas* reaches the maximum set by the user, the execution is stopped by launching an exception and the user who launched the execution loses the *gas* multiplied by the unit price. In case the execution ends, after paying the price of the used *gas*, the remaining *ether* will be returned to the user. The miners on their part receive a fee to reward them for maintaining the network. Their fees are defined by the *gas* and the price of the *gas*. If an attacker tries to launch a denial of service attack and chooses a price of *gas* according to the market, the miners will execute the attack but the price of the attack will be very high. On the other hand, if the attacker chose a very low text price, the miners would not include their transaction in any block and therefore the attack would not be executed.

### 2.3. Solidity

Smart contracts can be written in different high-level programming languages, Solidity being the most used [20]. Solidity is a high-level, object-oriented programming language for the implementation of smart contracts. It is characterized by having a strong influence of C++, Python, and JavaScript languages. This language is compiled into a bytecode that will be executed by the Ethereum Virtual Machine (EVM). Besides, it is a static typing language and goes through a constant process of updates that aim to minimize bugs and vulnerabilities. The first line (optional) of the smart contracts is *pragma solidity ˆx.x.x;* indicating the version of the Solidity compiler with which the contract was written to avoid undesired behaviour if it is compiled with a higher version (although for security reasons it is recommended to use the most recent versions of Solidity whenever possible). Smart contracts written in Solidity have a structure similar to classes in object-oriented languages and can inherit properties and methods from other contracts. Each smart contract can contain declarations of:

- **Status variables**: values that are permanently stored in the contract.
- **Functions**: units of executable code within a contract.
- **Function modifiers**: used to increase functionality and functions in a simple and declarative way.
- **Events**: communication interface with EVM logging tools for smart contract code debugging or alerts.
- **Structures**: these are custom data structures that can group several variables together.
- **Enumerations**: can be used to create custom data types that represent a finite set of status possibilities.

Algorithm 1 shows an example of a very basic smart contract.
**Algorithm 1** Smart contract example.1:pragma solidity ˆ0.4.0;2:contract SimpleStorage {3:    uint storedData;4:    **function** set(uint x) public {5:       storedData = x;6:    }7:    **function** get() public view returns (uint) {8:       return storedData;9:    }10:}

It can be seen that a global uint variable is declared within the contract and two getter and setter methods. Each time a user invokes this smart contract and assigns a new value, it is updated. Smart contracts can store state (as in the example), but it is not strictly mandatory and in fact, in some circumstances it may be better to have stateless contracts (dumb contracts). Although they are not used in this example, there are mechanisms in smart contracts to restrict access to certain functions depending on who executes the smart contract. An important fact to remember is that before adding a new smart contract to the blockchain, the smart contracts are executed locally in each mining node and the result of the execution must be deterministic to guarantee the coherence of the whole system. This property can be a problem when generating random numbers within smart contracts (as explained in the Section 3.1).

## 3. Security in Smart Contracts

Although the blockchain is securely designed and supported by widely studied and tested cryptographic algorithms, smart contracts such as software are likely to contain security vulnerabilities in their code, which given their immutable nature and coupled with the fact that they can operate in sensible domains like financial or health fields, poses a serious security threat, as has happened in the past.

### 3.1. Vulnerability Classification

The following is a description of the families of vulnerabilities in smart contracts. (In [21] a more detailed explanation of quite a few of them can be found):

- **Calls to the unknown**: It happens that some primitives of the Solidity language used to call other functions or send *ether* may suffer from a side effect of calling a function defined by default that all contracts have (and whose code could be unknown to the caller), in case the called function is not found. The primitives affected by this effect are:
  − *Call*: Is a primitive used to call the functions of a smart contract (of the same or another). If the function passed to the primitive as a parameter does not exist in the contract, a default function or fallback will be executed.
  − *Send*: This primitive allows the user to send *ether* from the contract in execution to another addressee. Once ether’s quantity has been shipped, the default function is executed in the target contract.
  − *DelegateCall*: This primitive one is very similar to CALL except that in this one, the context of variables of the caller contract is used.
- **Gasless sending**: Sending *ether* using the primitive SEND could result in a *gas* exhaustion exception if the recipient is a contract has a *fallback* function with a lot of code.
- **Exception disorder**: In Solidity there are several situations that can cause an exception to be triggered during execution, namely: if the execution of the contract runs out of *gas*, if the call stack is exhausted, or if the exception is launched explicitly by calling the command *throw*. However, Solidity does not treat exceptions in the same way if they occur during a function call directly or using the primitive CALL. In the first case, the execution is stopped and any side effects are reversed, including *ether* transfers. However, if the exception occurred in the context of a call using CALL, the exception will propagate “up” by reversing the effects in the called contracts until the CALL call is reached, returning *false* and continuing the execution from there and consuming *gas*. This inconsistency in handling exceptions can lead to vulnerabilities.
- **Type conversion**: Although the Solidity compiler can detect errors with types, for example if a function waits for an integer and is called by passing it a string, in the case of contract definitions or functions with a certain structure, or in the case of calling a function in a contract, if the programmer makes a mistake and calls another contract by mistake but it contains a function with the same structure expected by the compiler, the function will be executed and if the function does not exist, the function of *fallback* will be called. In any case, no exception will be launched.
- **Re-entrancy**: This is a vulnerability well known for its impact. The programmer may think that a non-recursive function cannot be re-called while it is running, but this is not always the case, as it could be the case that within the function, an empty malicious contract is called that only contains a function of *fallback* that calls back the function it comes from. For example, suppose we have such a contract, as shown in Algorithm 2.
  The function *foo* receives, as a parameter, the address of a contract, and if the token is not activated, it sends 1 *wei* (ether’s minimum unit) to the contract c. After receiving the ether, Alice calls her *fallback* function, which in turn calls Bob’s *foo* function again and as the witness is not set to *true* it will transfer back to Alice *ether* and this will be repeated until the *gas* is exhausted or the call stack limit is reached. This vulnerability was used in the Decentralised Autonomous Organization (DAO) attack discussed in Section 3.3.
**Algorithm 2** Re-entrancy vulnerability example.1:contract Bob {2:      bool sent = false;3:      **function** foo(address c) {4:           if (!sent) {5:                  c.call.value(1)();6:                  sent = true;7:           }8:      }9:}10:contract Alice {11:      **function()**{12:           Bob(msg.sender).foo(this);13:      }14:}
- **Secrets**: Solidity allows you to define the visibility of fields in contracts as public or private. This can be useful if you need to hide certain information between contract calls. Unfortunately this system is not effective as changes in private fields have to be sent to mining nodes to be put into the blockchain, which is public.
- **Unpredictable state**: All smart contracts have a state determined by the value of their fields and their *ether* balance sheet. But you cannot guarantee that the state a contract had when we made a transaction to it will be the same as when that transaction is mined and included in the blockchain. That is to say, it could happen that before processing our transaction, other transactions have changed the status of the destination contract, and besides being fast, does not guarantee us anything because the miners can mine the transactions in the order they want. There is another problem added by the nature of the blockchain, and that is that a chain *fork* could occur if two miners continue to mine a valid block at the same time. This would cause some miners to try to add their block in one of the two strings and the others in the other. At some point the shorter chain would be discarded, losing the transactions contained in it and changing the state of the contracts to an indeterminate state. Another case would be contracts that use dynamic libraries (a special type of contract that cannot have mutable fields). Such contracts could maliciously change to deceive the victim who would call them without knowing that they have changed.
- **Random numbers**: The execution of the Ethereum virtual machine code is deterministic. This means that the code executed with the same inputs must produce the same output in all the nodes that execute it. This presents a problem when generating random numbers. To simulate randomness, many contracts use a random number generator initialized with the same seed for all miners. One option widely used by programmers is to use as seed the hash of a given block in the future. Being an unpredictable value a priori, it is a good way to initialize the generator of random numbers. However, since miners can choose which transactions to put in the new blocks, they could conspire to try to alter the operation of the random number generator.
- **Time restrictions**: Many applications have time restrictions to operate. Usually these restrictions use timestamps. In the case of smart contracts, the programmer can get the timestamp of when the block was mined, which is shared by all transactions in the block. The trouble is that miners in the early versions of the protocol could choose the timestamp of the block they were going to arbitrarily mine, which could be used to carry out attacks.
- **Immutable bugs**: Although This is not a vulnerability in itself, but the consequence of a blockchain property. All the source code of smart contracts, including those containing *bugs*, are immutable once they are mined and added to the blockchain, although they can be blocked by calling a destructor function.
- **Loss of ether**: If the programmer enters an address to send *ether* and that address exists but it is an orphan address that belongs to no one that *ether* will be lost forever.
- **Stack size**: Each time one contract calls another contract the associated call stack increases by one. The stack limit is 1024 and when the limit is reached an exception is launched. Until October 18, 2016 it was possible to take advantage of this to launch an attack where a malicious user increased the battery counter until almost exhausted and then called the victim’s function which launched an exception when the battery limit was exhausted. If the victim did not take this into account and does not handle the exception correctly, the attack could be successful. The impact of this vulnerability caused Ethereum to be redesigned.

### 3.2. Analysis Methods

- **Static analysis**: Static software analysis is a way of studying the behaviour of a program from its compiled binary code without executing it, looking for known patterns that often lead to vulnerabilities. There are several procedures when making a static analysis, like:
  − *Disassembly*: The process by which binary code is translated into an human-readable set of instructions.
  − *Decompilation*: The process to inversely generate from code disassembled code in a high-level language.
  − *Symbolic execution*: Symbols are used instead of using specific values for variables. Operations on these symbols lead to algebraic terms, and conditional statements give rise to propositional formulas that characterize branches. A particular part of the code is attainable if the conjunction of formulas on the way to this part is satisfactory, which can be checked by SMT solvers.
  − *Control flow graph*: The representation using a graph of the possible paths that the execution of a program can follow.
  − *Pattern recognition*: This searches for portions of bytecode known to contain potentially vulnerable code.
  − *Rule-based analysis*: A method that transforms the CFG into a representation of rules.
- **Dynamic analysis**: This type of analysis acts in the execution phase of the program, detecting vulnerabilities that could have gone unnoticed during the static analysis (for example due to the existence of obfuscated code or resistant packaging) at the same time that it serves to verify those found by this one.
  − *Execution trace*: As with the CFG in the static analysis, the trace of contract execution is generated by this during the execution.
  − *Symbolic analysis*: From the different traces at execution time, a symbolic execution of the contract is carried out by inserting different symbolic values in the variables and recording the different outputs produced.
  − *Validation of false/true positives*: After the symbolic analysis and once a contract has been identified as a possible vulnerable candidate, the result of the symbolic analysis is validated with concrete data.
- **Formal verification**: Through this type of analysis, a verification is carried out using formal mathematical methods to test the specific properties of the code, using theorems provers like SMT Z3 and Coq, among others.

### 3.3. Most Relevant Attacks on Smart Contracts

This section covers two security attacks on smart contracts that became famous because of their economic impact and relevance. Smart contracts usually handle money (usually in the form of cryptocurrencies), so they are a very lucrative target for cybercriminals. Due to the immutable nature of the blockchain, if a smart contract contains some vulnerability it will not be possible to patch it. It would be possible to "destroy" the smart contract (marking it as unusable) so that it cannot be executed, but any smart contract that depends on it will be affected, as shown in this section. It is therefore vitally important to test the source code of smart contracts before adding them to the blockchain and using a secure development methodology. For a more detailed explanation of these and other attacks, see [21].

#### 3.3.1. The DAO Attack

In 2016, 3.6 M Ether (ETH) was stolen (equivalent at the time to about $70 M) from a Decentralised Autonomous Organization (DAO). To do so, it took advantage of a re-entrancy Section 3.1 type vulnerability in a smart contract that managed the DAO’s funds, by means of which the attacker managed, with successive recursive calls to the method responsible for sending money to his account, to completely empty the DAO’s reserves. Algorithm 3 shows an example of the DAO’s smart contract with the aforementioned vulnerability and another example of a malicious contract that exploits the vulnerability present in the first smart contract.

**Algorithm 3** Vulnerable Decentralised Autonomous Organization (DAO) smart contract example.
1://DAOexample.sol2:contract DAOexample {3:    mapping(address => uint256) public funds;4:    **function** incrementUserCredit() payable {5:       funds[msg.sender] += msg.value;6:    }7:    **function** getUserCreditAmount() returns (uint) {8:       return funds[msg.sender];9:    }10:    **function** recoverUserCredit(uint amount) {11:       if (amount <= funds[msg.sender]) {12:            msg.sender.call.value(amount)();13:            funds[msg.sender] -= amount;14:       }15:    }16:}


Before continuing with the explanation of the source code of the smart contracts of the Algorithms 3 and 4 it must be explained to the reader that all smart contracts have a number of common variables, a very important one being ether’s balance sheet. This turns the default smart contracts into a kind of "piggy bank" where anyone can deposit money, but only specific users, and under specific conditions, can take it out. This is the case of the *DAOexample*. The contract *DAOexample* is a kind of bank that allows any user to deposit money and retrieve it later. To find out how much money each deposited user has in a bank, an associative array is used that maps an Ethereum address with an unsigned 256-bit integer counter. To prevent a user from trying to withdraw more money than he has deposited, line 11 checks the amount to withdraw with the value of the counter in the array. If the amount is less than or equal to the total funds deposited by the user in the *DAOexample* contract, the *call.value()* method of the contract that originated the execution of the *DAOexample* smart contract (which in our example is the DAOattack contract) is called. It is in that call to an external contract, where the re-entry vulnerability can occur, as happened in this case. First the attacker sends an amount to the contract *DAOexample* to be included in the registry. He then requests to recover the amount by calling the *recoverUserCredit* function which will transfer the amount to the *DAOattack* contract by executing the FALLBACK function of that contract, which will then call the *recoverUserCredit* function again. As the first call to *recoverUserCredit* has not updated the attacker’s balance, it will transfer the same amount to the attacker, repeating the whole process and thus, by means of successive recursive calls, emptying the funds of the *DAOexample* smart contract. In line 15 of *DAOattack* the attacker finally transfers all funds stolen from *DAOexample* (and currently in *DAOattack*) to the owner address that controls the attacker. There are three Etehreum addresses involved in the attack, the *DAOexample* smart contract address, the *DAOattack* smart contract address, and a user address that controls the attacker.
**Algorithm 4** Smart contract used to attack DAO example.1://DAOattack.sol2:import ’DAOexample.sol’3:contract DAOattack {4:    DAOexample public dao = DAOexample(0xAAAAA…);5:    address owner;6:    **constructor**(DAOattack) public {7:       owner = msg.sender;8:    }9: 10:    //FALLBACK FUNCTION11:    **function**() public {12:       dao.recoverUserCredit(dao.getUserCreditAmount(this));13:    }14:    **function** stealFunds() payable public{15:       owner.transfer(address(this).balance);16:    }17:}

Some of the solutions to avoid this:

- Swap the lines 12 by 13 in *DAOexample* so that the ether balance of the attacker is first updated and in the next recursive call the condition is false as seen in Algorithm 5.
- Use the methods *send()* and *transfer()* to send money to another contract instead of *call.value()*. The reason is that the methods send() and transfer() have by default limited *gas* consumption to a very low value that would not allow the successive recursive calls needed to perform this attack.
- Use some kind of *mutex* object in order to protect the sensible parts.

This attack had such an impact that it divided the global Ethereum community into two groups. Those who considered it necessary to reverse the fraudulent transactions of the DAO attack (remove them from the blockchain) and those who argued that the principle of the immutability of the blockchain should prevail above all in any circumstance. This division produced a *hard fork* of the Ethereum’s blockchain giving rise to a new branch of Ethereum and the old branch was renamed Ethereum Classic (where the funds stolen by hackers are still valid). The DAO attack also marked a turning point in the development of Ethereum and serves as a reference to teach developers the consequences of sloppy programming while encouraging the entire community to carefully test all the source code of contracts that are added to the blockchain. (A more detailed explanation of this attack can be found in [22]).
**Algorithm 5** Non-vulnerable DAO smart contract example.1://DAOexample.sol2:contract DAOexample {3:    mapping(address => uint256) public funds;4:    **function** incrementUserCredit() payable {5:       funds[msg.sender] += msg.value;6:    }7:    **function** getUserCreditAmount() returns (uint) {8:       return funds[msg.sender];9:    }10:    **function** recoverUserCredit(uint amount) {11:       if (amount <= funds[msg.sender]) {12:            funds[msg.sender] -= amount; *//Update balance before the external call*13:            msg.sender.call.value(amount)();14:       }15:    }16:}

#### 3.3.2. Parity Multi-Signature Wallet (Double) Attack

In 2017 there was an attack on parity multi-signature wallets in Ethereum. These attacks are a little more sophisticated than the DAO (but not much more either). Before explaining the attack further, it must be explained that a multi-signature wallet is a type of smart contract that allows a user to manage their funds using different private keys, different limits, and so on. To facilitate the programming of these smart contracts, most of the logic and functions are reused through a library (another smart contract) of public access. Algorithm 6 shows a simplified version of Parity wallet.

The attacker started the attack by sending a transaction to *walletExample* requesting to execute the *initWallet* method. As the *walletExample* contract did not have this method, the FALLBACK method was skipped, which by means of *delegatecall* called the *initWallet* method of the library contract (which was not protected against external calls). In this way, the attacker became the owner of the victim’s wallet and withdrew all the funds it contained. The attacker repeated this process with more wallets affected and managed to steal $31 M. On this occasion, a hard fork, as happened in the DAO attack, made no sense because there was no reaction time (the DAO smart contract had an implemented delay of one month before allowing hackers to withdraw the funds, giving the community time to decide what to do). What happened in this case is that a group of white-hat hackers went ahead of the attackers, identifying the wallets affected by this vulnerability and emptying them before, transferring the funds to a secure wallet. From then on, the money was returned to its owners and the vulnerability was corrected. However, the first stolen amount was lost forever. A more detailed explanation of this attack can be found in [23].
**Algorithm 6** Simplified version of vulnerable Parity multi-signature wallet.1:contract walletExample {2:    address walletLib = new walletLibExample();3:    address owner;4:…5:    //FALLBACK6:    **function** () payable {7:       if(msg.data.lenght > 0) {8:            walletLib.delegatecall(msg.data);9:       }10:    }11:}12: 13:contract walletLibExample {14:    …15:    **function** initWallet(address[] owners, uint required, uint daylimit) {16:       initDaylimit(daylimit);17:       initMultiowned(owners, required);18:       }19:    }

A possible solution to this attack is to add a variable that controls whether the wallet has already been initialised. This was done by the developers of Parity after the attack, but they made the mistake of not initialising the shared library, which was taken advantage of in another attack that, this time, could not access the funds of the wallets, but eliminated the library calling the *suicide* method and leaving all the wallets blocked that depended on this library, freezing approximately $280 M forever.

## 4. Tools

This section presents a list with brief commentary of several open source security tools for smart contracts in chronological order of emergence. To test all the tools, a virtual machine (four cores@8GB RAM) with Ubuntu 18.04.3 LTS and kernel 4.15.0-72-generic has been used. The testing methodology that has been systematically followed was to download the source code of the tools from their respective public repositories. After that, all the necessary dependency packages were installed and each tool was compiled. After that, several runs with example files of the tools were made to check their functionality.

### 4.1. Oyente

**Figure 2:**
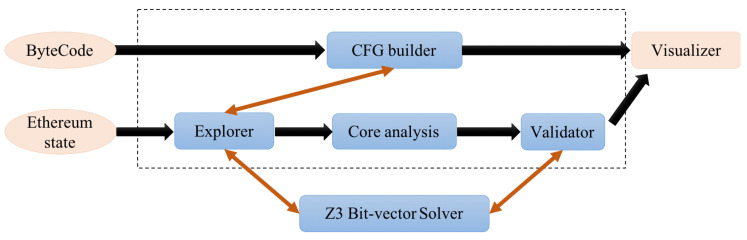
Oyente operating scheme.

Oyente was one of the first smart contracts security tools to emerge [24]. It executes the EVM bytecode of smart contracts in a symbolic way and focuses on checking blocks of code likely to be vulnerable because of their structure, calls to external contracts, or poor management of execution exceptions. Figure 2 displays its operating scheme. It is available on Github [25] from January 2016 (GPL-3.0 licence). Oyente’s origins come from a Ethereum Git Repository that has been moved to a new location, called MelonProject. Oyente has serious dependencies on Python 2 and 3, EVM from Go-Ethereum [26], and the Solidity compiler, and because of this, it was impossible to make a full installation of each dependency, forcing the analysis to a docker image. Oyente is a symbolic execution tool working with EVM bytecode directly, offering a way to research breaches in contracts deployed in Ethereum. Indeed, it is a great feature because the Ethereum blockchain only stored smart contract bytecodes. Mainly, Oyente could find among others the following vulnerabilities:

- Parity Multi-signature Bug.
- Callstack Depth Attack.
- Timestamp Dependency.
- Re-entracy problem.

**Figure 3:**
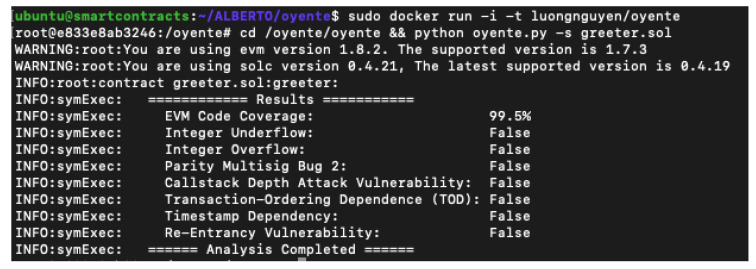
Oyente execution results.

Oyente is a really hard tool to install based on the fact that has many dependencies, a lot of them older versions of libraries. The documentation is very short compared to other tools, even then you could not find which vulnerabilities could be analysed. The best option to make a quick install is to use the docker that the owner provides. Besides the use of docker images, it has one main problem, the versions. Since the docker was created, the developers do not update the libraries, and due to this, the solc (Solidity compiler) version and EVM are not the latest. In Figure 3 oyente execution results are shown.

### 4.2. Remix-IDE

Remix-IDE is an extension for the web browser that allows you to write smart contracts in the Solidity language. It shows graphically different warnings of potential vulnerabilities or failures in the code at the same time as it makes a light static analysis. It is available on Github from April 2016 [27] (MIT license). This IDE enables a user to develop smart contracts in an incredible way. Is a browser-based IDE which provides the following features:

- Online editor to write contracts in Solidity.
- Solidity Compiler Switcher and EVM Version selector.
- Solidity Static Analysis.
- Plugin Manager.

**Figure 4:**
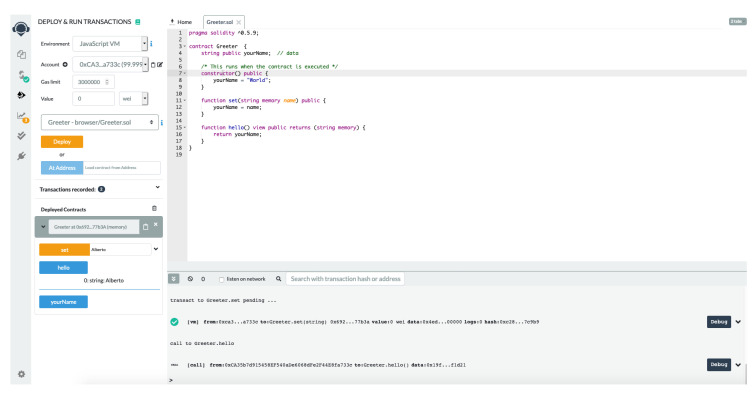
Remix-IDE: Online editor + Solidity compiler.

In Figure 4 shows the main window of Remix-IDE. One of the most powerful features that Remix-IDE gives to users is the chance to install plugins. It is important to notice that some plugins are vulnerabilities scanner tools such as Etherscan, Venom, GasProfiler, and more.

### 4.3. Solgraph

Solgraph is a tool that allows you to visualize the execution flow of a smart contract written in Solidity and to see potential vulnerabilities in an easier way. It is available on Github [28] from July 2016. The Solgraph tool generates a DOT file, containing a graph showing the function control flow of a smart contract writing in Solidity. A DOT file is a graph description language. It shows different actions represented with different colours:

- Red: Send to external address.
- Blue: Constant function.
- Yellow: View.
- Green: Pure.
- Orange: Call.
- Purple: Transfer.
- Lilac: Payable.

After that, it is needed to transform a .dot file into a .png. The tool is written in JavaScript as an npm package. It is easy to install onto every system running Node.js and an npm manager.

### 4.4. MadMax

MadMax is an extension of the Vandal tool, allowing the possibility to find gas vulnerabilities [29]. The tool analyses the EVM bytecode the same way as Vandal (it has the same options and no extra library is required to work). MadMax gives:

- The use of static EVM bytecode decompiler, mainly because of the problem with the stack-based low-level nature and the low control-flow structures (that were discussed before).
- The ability to find gas vulnerabilities.
- An abstraction to understand EVM bytecode by giving a high-level data structure to this level and making an approach between EVM low-level and high-level vulnerabilities.
- The ability to validate a huge number of contracts already deployed on the Ethereum blockchain.

**Figure 5:**
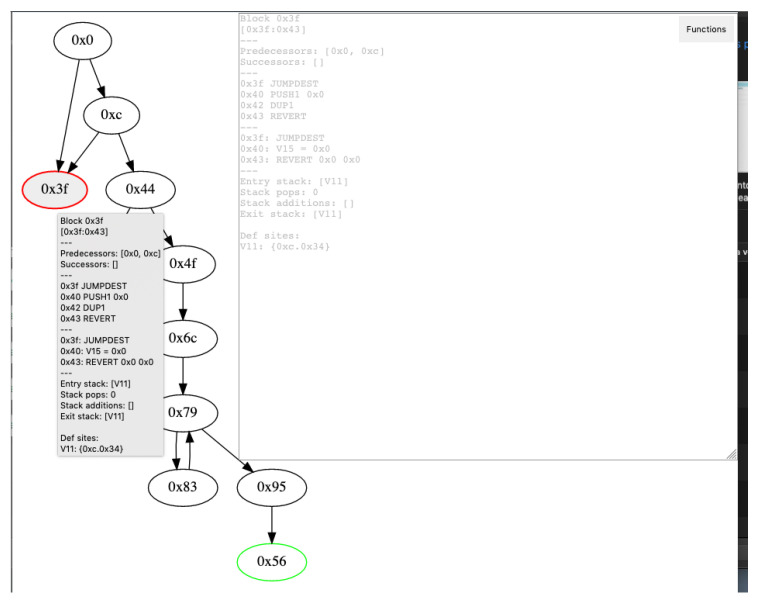
MadMax results.

Performing in the same way that Vandal, the decompiler works by giving the possibility to construct a graph diagram. At this point, if the following command is running, the graph will be generated for a contract bytecode. Using the smart contract in Algorithm 7 will generate the results shown in Figure 5 (notice the integer overflow vulnerability due to uint max size and the initial *i* value):
**Algorithm 7** Loop smart contract.1:pragma solidity ˆ0.5.0;2:contract TestLoop {3:    **function** test() public returns (uint) {4:       uint x = 0;5:       for (uint i = 0; i < 256; i++) {6:            x = x*i + x;7:       }8:       return x;9:    }10:}

The control flow diagram provides a good way to understand the different calls, jumps, and how the stack works. Furthermore, the top "functions" button enables you to filter the nodes by public, private, fall-backs, etc.

### 4.5. Manticore

**Figure 6:**
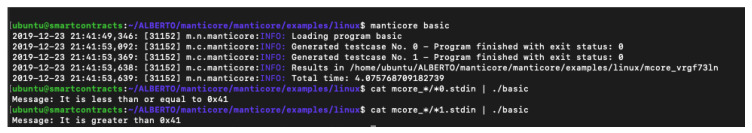
Manticore execution output.

Manticore is a symbolic execution tool meaning that the way it works is by analysing all the paths that one contract could take [30]. This class of analysis has some limitations, the most notorious is the difficulty in discovering vulnerabilities in huge contracts. Contract bytecode and Linux ELF binaries are the two types of files that Manticore can work with. The tool has the capacity to generate inputs, trigger them, trace the execution, and capture bugs  [31]. It is available on Github [32] from February 2017 (AGPL-3.0 license). Manticore is really simple to install, having some dependencies on Python 3. After the installation process, the user can execute an example running test. Besides, it has a Python API, which contains different Python scripts that launch specific functions, depending on what the user wants to get. In Figure 6 you can see the output of running manticore on an input example C program in Algorithm 8. The basic files contain a small program that checks if a binary packed integer at the input is 0x41 or less. Then, the tool will generate a folder with the basic file test cases.
**Algorithm 8** Manticore example C program.1:#include <stdio.h>2:#include <stdlib.h>3:#include <unistd.h>4: 5:int main(int argc, char **argv) {6: 7:    unsigned int cmd;8: 9:    if(read(0, &cmd, sizeof(cmd)) != sizeof(cmd))10:   {11:           printf("Error reading stdin");12:           exit=1;13:   }14: 15:   if(cmd > 0x41)16:   {17:           printf("Message: it is greater than 0x41");18:   } else {19:           printf("Message: it is less than or equal to 0x41");20:   }21: 22:   return 0;23:}

Notice that Manticore could also perform an analysis of .sol extensions files, not just bytecodes files. The API functions just work with bytecode files.

There is a public wiki with all API functions explained: https://manticore.readthedocs.io/en/latest/.

### 4.6. SmartCheck

**Figure 7:**
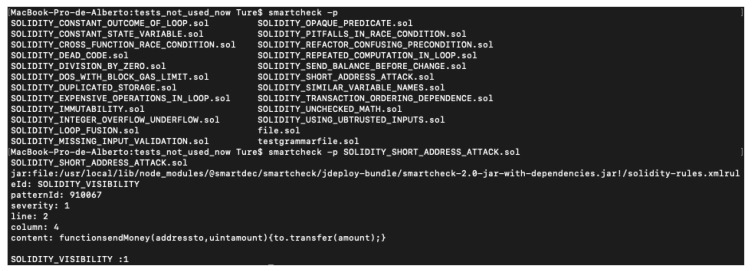
SmartCheck: Solidity_short_address_attack.sol analysis.

SmartCheck looks for known vulnerability patterns in the source code [33]. It uses an XML file with the source code in the form of a tree and explores paths that can lead to vulnerabilities during execution. Available on Github [34] from May 2017 (GPL-3.0 license). Smartcheck could detect many vulnerabilities, working really efficiently, showing where the possible vulnerability is, based on predefined rules. Even though Smartcheck could be installed as an npm package, it also has an online website, where it is possible to easily upload smart contracts. SmartCheck provides a small library to check different vulnerable contracts. Figure 7 shows a short address vulnerability after it runs an example library contract. By default, Smartcheck has some types of severity for each vulnerability, showing the line, pattern identifier, line, column, and content.

### 4.7. Mythril

Mythril is a tool very similar to Manticore, which allows the symbolic execution of the EVM bytecode and generates a flow control graph [35]. All this makes it possible to detect a series of types of vulnerabilities. Developed by the company ConsenSys, is available on Github [36] from September 2017. As Mythril Github says, it is a security analysis tool for EVM bytecode. It can perform in two ways, analysing .sol extension files and giving, as a parameter, the contract address from the Ethereum blockchain.

On the capabilities analysis side, Mythril has a directory allocating the different modules. Each module contains a specific kind of vulnerability to find. Modules are:

- Delegate Call Untrusted Contract.
- Delegate Call To Untrusted Contract.
- Dependence on Predictable Variables.
- Deprecated Opcodes.
- Ether Thief.
- Exceptions.
- External Calls.
- Integer.
- Multiple Sends.
- Suicide.
- State Change External Calls.
- Unchecked return value
- User Supplied assertion.
- Arbitrary Storage Write.
- Arbitrary Jump.

**Figure 8:**
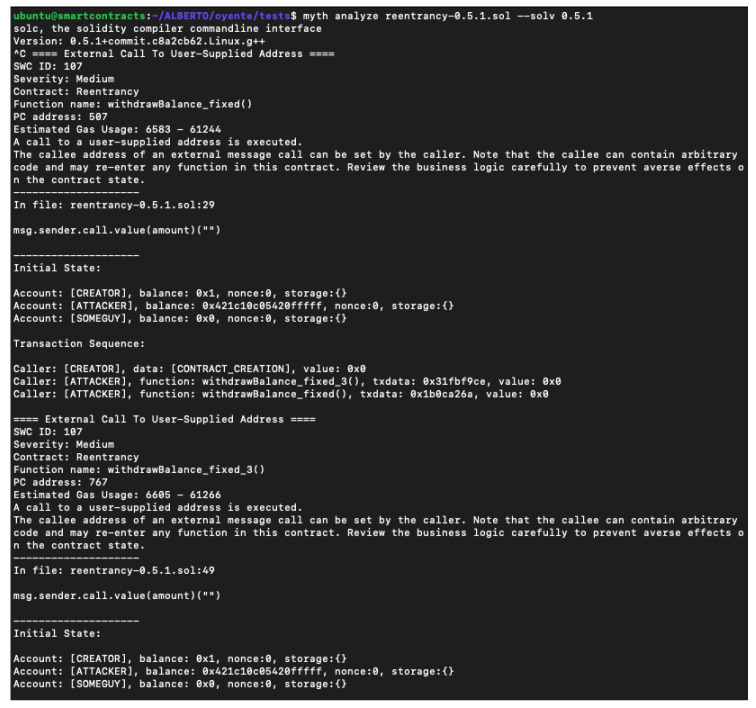
Mythril: partial analysis results.

The tool is prepared to be module-extensible, anybody could create their own module. The installation process consists of downloading a docker image or installing a Python 3 module. More documentation could be found here: https://mythril-classic.readthedocs.io/en/master/. As can be seen in Figure 8, the amount of information is impressive. Clarifying the analysis process, in the first place there is a summary of analysis, giving advice of what an attacker could do to exploit the bug. Furthermore, gas estimation, severity, and the vulnerable function name are shown. The initial state and transaction appears too, with different test cases for each function.

### 4.8. ContractLarva

ContractLarva is a tool that verifies smart contracts at runtime [37]. To do this, when compiling the contract, it adds to the original source code a series of instructions to ensure during the execution that vulnerabilities do not occur and/or avoids them as far as possible without altering the normal flow of execution of the contract. It is available on Github [38] from December 2017 (Apache-2.0 license). One of the major problems found trying to compile the tool was the installation in Ubuntu following the standards. Notice that ContractLarva uses a Haskell version with some functions that the default Ubuntu package installation does not have. The tool can be invoked by writing at the command line: *contractlarva pecification.dae input.sol output.sol*.

- Specification: the properties to capture the events. ContractLarva allows capturing just two types: control flow events (entry and exit function points) and data flow events corresponding to changes in values of variables.
- Input: the input contract.
- Output: results of analysis.

DAE structure could be found in the documentation.

### 4.9. SolMet

SolMet is a tool to calculate the complexity of the source code of a smart contract written in Solidity that uses a parser to generate an abstract syntax tree where it evaluates a series of metrics to measure complexity [39]. It is available on Github [40] from February 2018. SolMet allows the user to perform a static analysis. It performs this analysis using its own metric calculator tool for Solidity, composed by the following metrics:

- SLOC—number of source code lines.
- LLOC—number of logical code lines (lines without empty and comment lines).
- CLOC—number of comment lines.
- NF—number of functions.
- McCC—McCabe’s cyclomatic complexity.
- WMC—weighted sum of McCabe’s style complexity over the functions of a contract.
- NL—the deepest nesting level of control structures in functions summed for a contract.
- NLE—nesting level else-if.
- NUMPAR—number of parameters.
- NOS—number of statements.
- DIT—depth of inheritance tree.
- NOA—number of ancestors.
- NOD—number of descendants.
- CBO—coupling between object classes.
- NA—number of attributes (i.e., states).
- NOI—number of outgoing invocations (i.e., fan-out).

**Figure 9:**
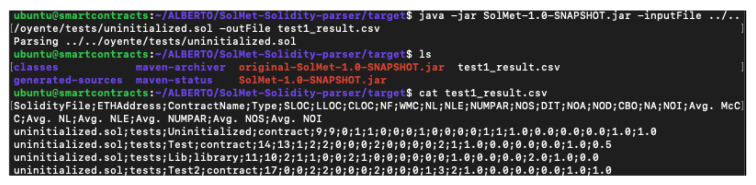
SolMet: analysis results.

All this information could be find in the git repo: https://github.com/chicxurug/SolMet-Solidity-parser To use the tool, it is required to install the Maven package because the execute file is a .jar. One of the problems using a Ubuntu VM is opening, in a clear way, files like this test. After testing the tool with *uninitialized.sol* smart contract, the results in the command prompt are shown in Figure 9.

As is seen, all metrics listed before are there, illustrating the differences between library, contract, and each metric. This is a nice tool to determine the insights of a data-sheet of smart contracts.

### 4.10. Vandal

Vandal is a Solidity disassembler and decompiler that generates a flow control graph that can be viewed as an HTML web page [41]. It also allows you to specify the security analyses in a logical way using the Soufflé language [42]. It is available on Github [43] from February 2018. Vandal is a powerful tool, being different from the others. Disassembling, decompiling, and analysing libraries using Datalogs are the three types of analysis which are available. All of them work with contract bytecodes. There is a lot of information about the tool in the Github wiki. Summarising:

- Disassembler (works with Python): Provides extra functionality compare to Ethereum one.
- Decompiler: The creators of the tool says that is the core of Vandal, converting EVM bytecode to an intermediate representation enabling the creation of a control flow diagram (just like the SolGraph program).
- Datalog: Vandal uses the Soufflé Datalog engine, detecting logic and different Solidity operation code to determine whether it is a vulnerability or not.

**Figure 10:**
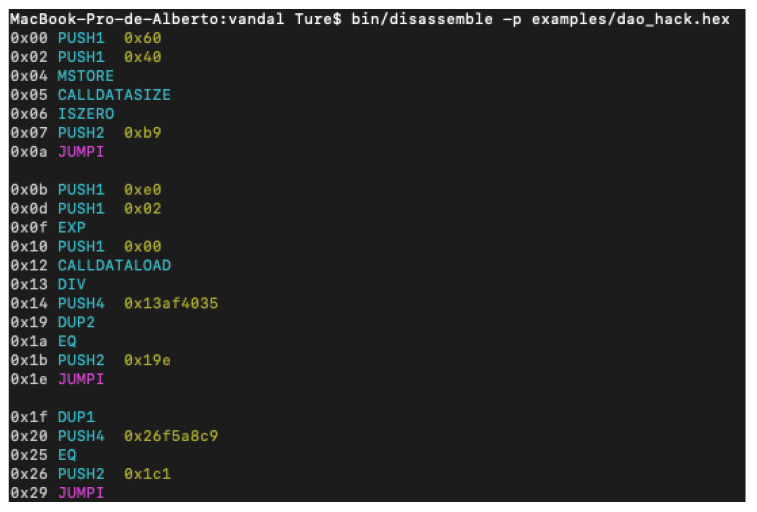
Vandal: disassembly.

Furthermore, repo provides a bulk analysis script to perform multiple analyses at once. At the beginning, for testing reasons, it was decided to run only the bulk script, which was the better way to perform fast testing, however, it was found that in two different systems (Mac Os X and Ubuntu) the script fails. In Figure 10, a disassembly of an vulnerable smart contract can be seen. The repository has opened issues since 2018. It seems to be stopped or abandoned.

### 4.11. EthIR

EthIR is a framework that transforms the source code of smart contracts into an intermediate language [37]. It is based on the Listener tool but uses a rule-based representation. It is available on Github [44] from March 2018 (GPL-3.0 license). EthIR is an evolution of Oyente. The basic commands are quite similar to the Oyente tool (because it is an extension of it), thus giving an extra value to Oyente by generating a rule-base representation of the program (contract). The purpose of this is to give an application of existing high-level analysis to add or infer properties to EVM code. To perform the rule-base representation (RBR), a disassembly file is needed. After running the script to generate the RBR command, the representation of values are just like we could find in git: https://github.com/costa-group/EthIR. The RBR produces a file to be utilised with other tools. In fact, this option gives a customization subprocess after the generation of the RBR, but on its own, the RBR file does not provide immediately useful information.

### 4.12. MAIAN

MAIAN is a tool very similar to Oyente, but is broader by taking into account the attacks that require several transactions [45]. It relies on the use of a private blockchain for testing and thus mitigates false positives. Like many other tools, it uses the SMT Z3 solver to look for execution paths that lead to potential vulnerabilities. It is available on Github [46] from March 2018 (MIT license). MAIAN is one of the easiest tools presented in this document. It is a static analyser for three types of major categories of Ethereum smart contracts:

- Prodigal: These leak funds to arbitrary users.
- Suicidal: These contracts can be killed by any user.
- Greedy: These contracts lock funds indefinitely.

Using a Python script, just run the tool. This has dependencies like most of the tools in this document, the Solidity Compiler (Solc), Go Ethereum, Z3 theorem prover, web3, etc. It was not possible to install the tool. A web3 dependency error did not disappear, even after being installed.

### 4.13. Erays

**Figure 11:**
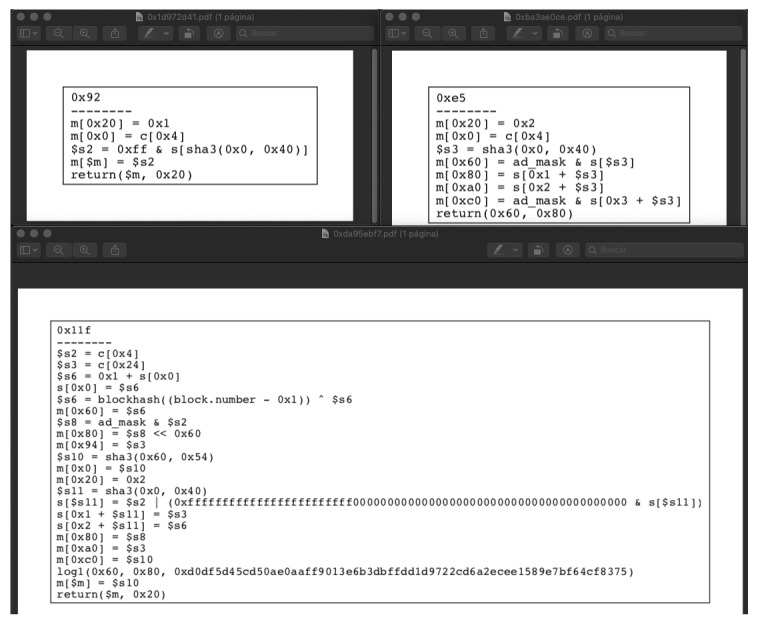
Erays: PDF results.

Erays is an EVM bytecode disassembler [47]. It allows the user to generate a PDF file with pseudocode of the routines present in the smart contracts. It is available on Github [48] from August 2018 (MIT license). As the Git description says, Erays is a reverse-engineering tool to make the bytecode reading process easy. The output is generated using graphviz, which must be installed. The tool needs just a Python script to run. Then, the PDF output will be placed into a temp file. The output file is a PDF extension in order to simplify the reading. Erays was tested running the .hex file from temp folder. After running it, five PDF files were output. Figure 11 displays three of them.

Each file contains the "decompilation" (the owner says that Erays is not a decompiler, but is a better way to understand hex files) from each function inside the input file.

### 4.14. Rattle

**Figure 12:**
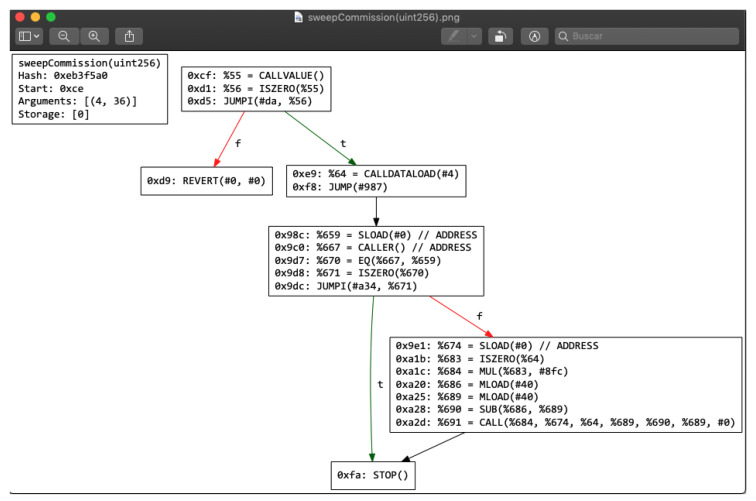
Rattle: analysis results.

Rattle is a framework that does binary static analysis of the EVM bytecode, performing a disassembly that eliminates instructions not necessary to understand the operation of the contract source code, although it does not detect vulnerabilities by itself [49]. It is available on Github [50] from August 2018 (GPL-3.0 license). One of the problems that EVM has is the difficulty of tracking and following variables due to the stack machine implemented on EVM. Rattle has the same purpose as Erays, giving a control flow diagram as output and trying to remove EVM operation codes to make the reading process easier, by transforming the stack machine into a Static Single Assignment form (SSA form). A Static Single Assignment form (SSA form) is the core of Rattle. SSA is an intermediate representation in compiler design, giving to each variable a value before it is used. The benefits of this conversion is to remove 60% of all EVM instructions. Therefore, Rattle tries to provide a way to trust unknown smart contracts by tracking the variables and making a contract auditable and reading-friendly at the same time. As it was said, Rattle has the same dependencies as Erays, being dependent on the same tools (Graphviz, Solc, and Python). Rattle works with runtime code. This creates the need to transform hex string files into bytes. As is seen in Figure 12, it is possible to check the control flow diagram of each function. To implement Rattle, it is recommended to read the following wiki in order to be able to understand the graph: https://www.trailofbits.com/presentations/rattle/.

### 4.15. Osiris

**Figure 13:**
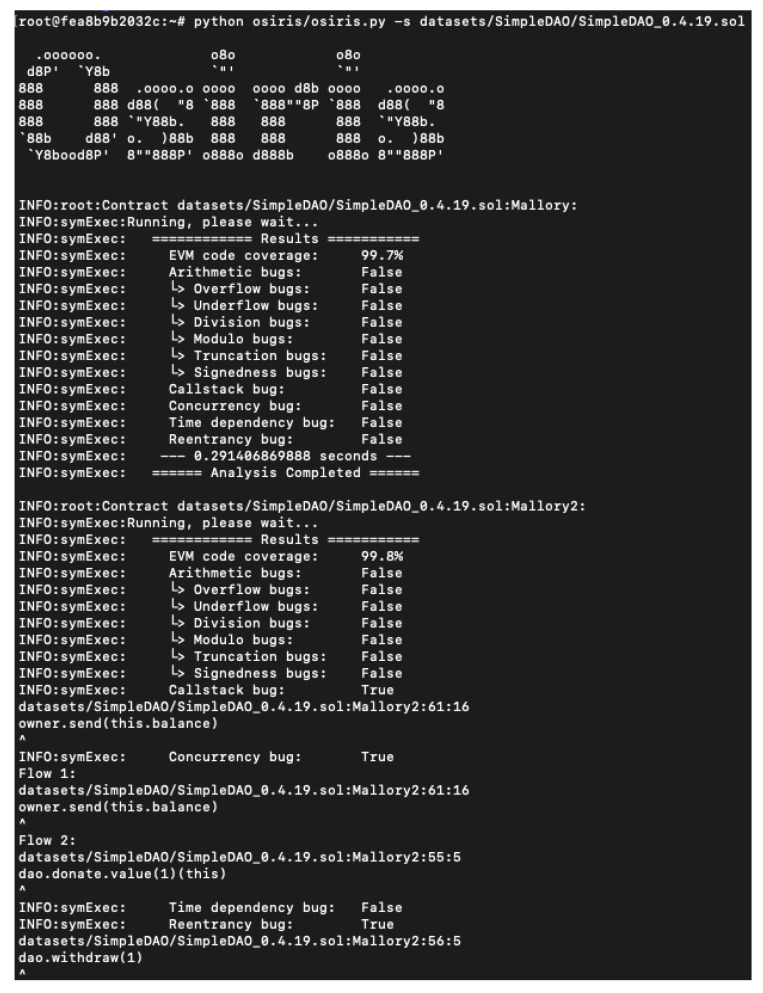
Osiris: analysis results.

Osiris is a tool specialized in detecting vulnerabilities related to integers within Ethereum’s smart contracts [51]. It works by extending Oyente’s functionality by analysing the execution flow to distinguish benign from malignant overflows. It is available on Github [52] from September 2018. Just like Oyente, it depends on the same libraries: web3, Z3 Theorem Prover (4.6.0 version), Python, and pysha3. Having the same troubles as Oyente, we were forced to use a docker image to be able to run the tool. The tool shows the output in the same way as Oyente, but checked different vulnerabilities. Furthermore, it shows a flow showing where the error could be and giving information about the vulnerability type. Figure 13 shows the analysis results.

### 4.16. Securify

**Figure 14:**
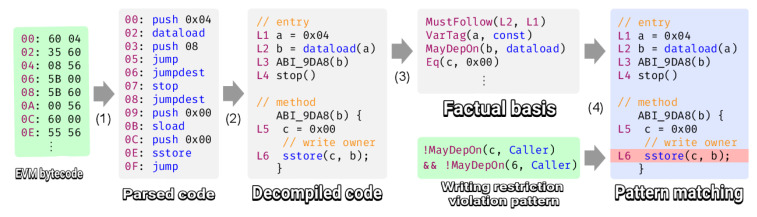
Securify code analysis [53].

Securify first performs a disassembly of the EVM bytecode [53]. Then, the disassembled code decompiles it into an intermediate language with which it creates a set of Datalog rules (as shown in Figure 14) that it will use to search for pattern violation and find vulnerabilities. Available on Github [54] from September 2018 (Apache-2.0 license). Securify offers an online GUI supported by Ethereum foundation at the same time that is possible to install the tool in a personal machine.

The analysis features that Securify offers are:

- Some forms of the DAO bug (also known as re-entrancy).
- Locked ether.
- Missing input validation.
- Transaction ordering-dependent amount, receiver, and transfer.
- Unhandled exceptions unrestricted ether flow.

**Figure 15:**
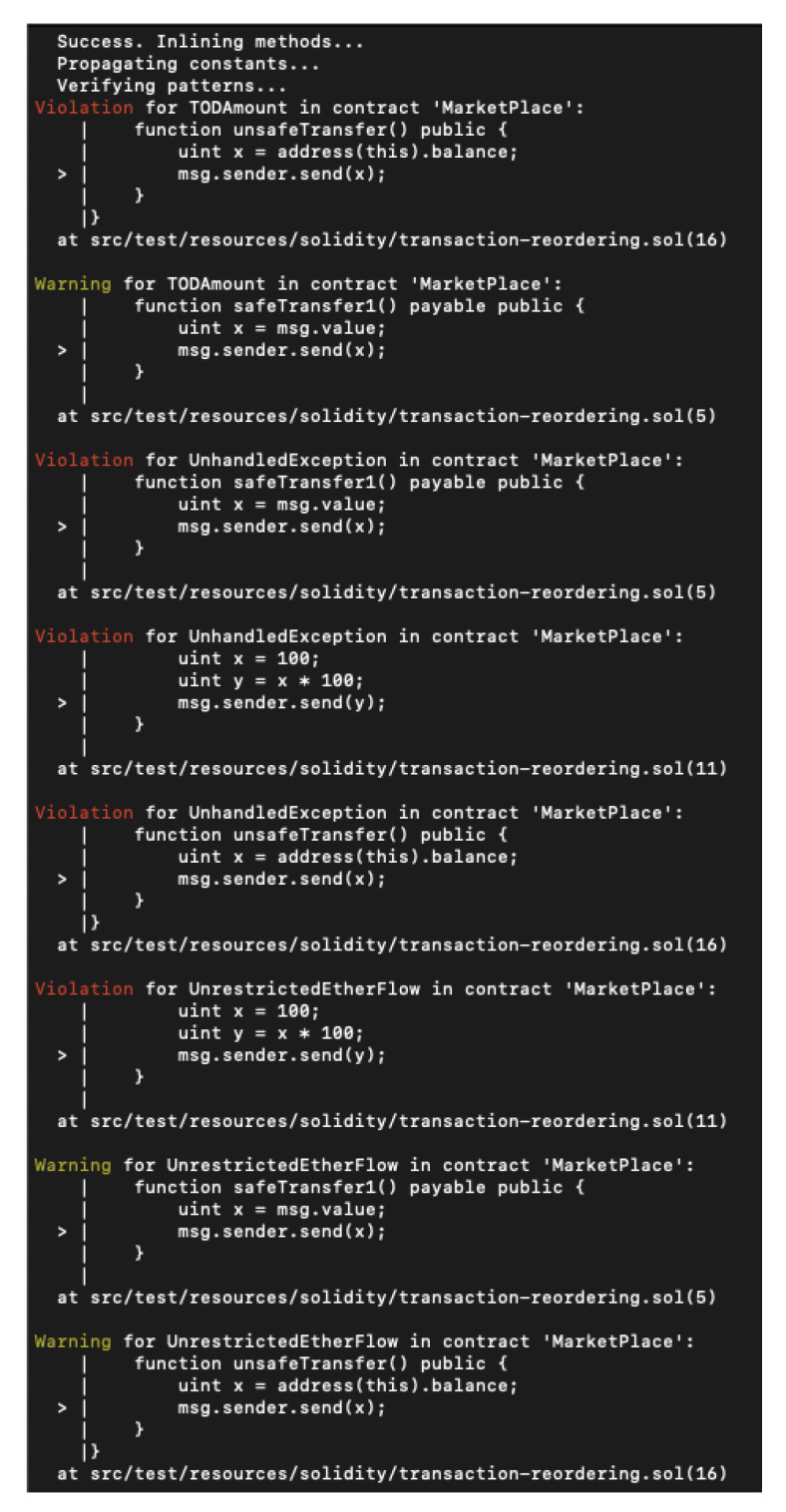
Securify analysis results.

Soufflé statically analyses the hex code of a smart contract. Then, Securify tries to discover violations or prove the compliance of security-relevant instructions. Securify needs the Java 8 library, Soufflé (same as Vandal), and Solc. The output file is a .json file. The report just shows warnings and vulnerabilities. If someone wants to add the compliance information, they need to use the –co flag. After running one of the basic test files that the Git owner provided, the analysis results are in Figure 15. As it was said before, it just shows warnings and vulnerabilities, in this case it was able to see the UnhandledException and the Unrestricted ether flow violation.

### 4.17. Slither

Slither is a static analysis framework written in Python 3 with dependencies on the Solidity Compiler (Solc) [55]. Slither uses a bundle of vulnerability detectors, printing the analysis results. It is available on Github [56] from September 2018 (AGPL-3.0 license). Some features of the framework are:

- Detection of low false-positives.
- The position of the errors in the code.
- Availability to analyse smart contracts from Solidity version 0.4.
- The partition of 99.9% public Solidity code.
- Average execution time of less than 1 second per contract.

The tool has 40 vulnerability detectors, each detector checks just one vulnerability (1:1 scenario). All the vulnerabilities are listed here: https://github.com/crytic/slither/wiki/Detector-Documentation. After the installation of Python 3 and Solc, the user can execute the tools running different commands in the terminal. Each command is based on crytic-compile, which is a library to help the compilation of smart contracts. Is integrated into Slither.

**Figure 16:**
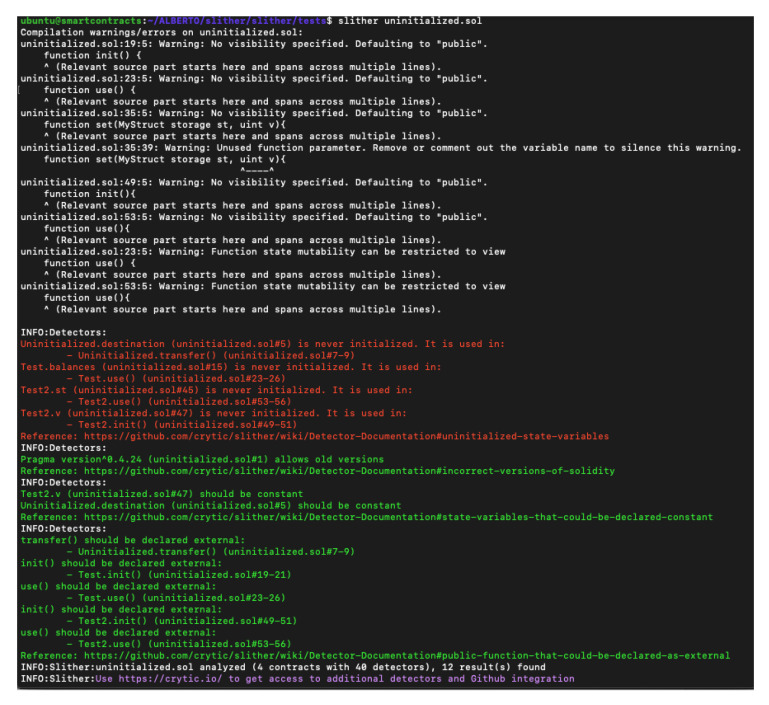
Slither analysis results.

By default, the Solidity compiler accepts contracts from the 0.6 version. To solve this problem, the user needs to install the “Sol-Select tool” which helps the user to switch between different Solidity versions. Slither has multiple configuration options, such as detector selection, printer selection, path filtering (filter the results by directory or filename), setting a configuration file to next executions, etc. One really good thing is the printer selection, providing a better way to understand the analysis. Below, a printer selection test is presented. As it was said before, Slither could perform in many different ways. To show a simple example a user just needs to run a contract stored in the test folder, see Figure 16.

### 4.18. EtherTrust

EtherTrust is a framework that bases its operation on translating the EVM bytecode smart contracts into Horn clauses, that, together with both *ether* and the SMT Z3 solver, verifies that the contract code does not present potential vulnerabilities, although it does not detect them [57]. It is available on Github [58] from August 2019 (GPL-3.0 license). EtherTrust Github has no readme file and the wiki is completely empty. At this point the best way to understand what the tool does is read the original survey: https://www.netidee.at/sites/default/files/2018-07/staticanalysis.pdf. They present the tool with the implementation of “the first sound and automated static analysis technique for EVM bytecode, which is practical and scales to large contracts”.

Besides, they compare the tool against Oyente, which seems to have better results: “We develop EtherTrust, a static analyzer that internally relies on the Z3 theorem prover for discharging proof obligations. We tested EtherTrust on a benchmark suite collecting code snippets from the literature as well as from real-life contracts stored on the blockchain, and compared its performance against Oyente. EtherTrust analyses large contracts in a few seconds, outperforming Oyente in coverage and efficiency by one order of magnitude. Furthermore, it also offers better precision in our benchmark, all of that despite being the first tool in the literature to provide formal security guarantees for EVM bytecode”. In real terms, the tool, if a proof-of-concept, does not detect vulnerabilities, but checks properties like: independence from transaction environment, single entrancy, and the rejection of the apparition of some.

To run the tool, first compile the Z3 Theorem Prover, get the com.microsoft.z3.jar package, and add it to the path EtherTrust/lib. After this point, unfortunately there was no documentation provided to make any kind of analysis.

### 4.19. Summary

Table 2 shows a short list of the specifications of each tool. On the other hand, Table 3 summarizes the process of installing and launching all the tools. Column headers are selected, considering the real application of each tool, outside of the theoretical and hypothetical world. The range selected for the categories Ease of Installation and Usefulness are 1 to 5. The scale is: 1—hard installation/useless to 5—easy installation/full utility.

## 5. Conclusions

We believe that security of smart contracts are topical fields of research. Although there are currently a relatively large number of tools available to developers, we believe that precisely that variety of options can confuse and make it difficult as to where to start working with them. Documents like this could serve as a starting point. Specifically, in relation to vulnerabilities in smart contracts it can be seen that two of the biggest attacks in terms of economic impact are relatively simple, technically speaking, but in the case of smart contracts, not only must we be careful when writing the code, but the distributed and immutable nature of the blockchain that makes any small error have unpredictable consequences needs to be considered. With a security-oriented development methodology and adequate testing tools (we believe that much work remains to be done in this field), this type of failure could be greatly reduced in the future.

## Figures and Tables

**Table 1:** DLT strengths vs. weakness summary [17].

	Blockchain	Tangle	Hashgraph
	- Transparency	- No mining required	-High number of transactions
Strengths	- Lots of implementation	- No transaction fee	- Scale in number of transactions
	- In production	- Quantum security	- Fairness
	- Small block size	- Single organization	- Gossiping overhead
	- Low scalability	management	- Use coins toss to terminate
Weakness	- Low processing speed	consensuns	
	- Lack of interoperability		- Permissioned network
	- Transaction fees		

**Table 2:** Summary of security tools specifications.

		Oyente	Remix-IDE	Solgraph	MadMax	Manticore	SmartCheck	Mythril	ContractLarva	SolMet	Vandal	EthIR	MAIAN	Erays	Rattle	Osiris	Securify	Slither	EtherTrust
Nivel	Bytecode	✓	✗	✗	✓	✓	✗	✓	✗	✗	✓	✓	✓	✓	✓	✓	✓	✓	✓
	Solidity	✗	✓	✓	✗	✗	✓	✗	✓	✓	✗	✗	✗	✗	✗	✗	✗	✓	✗
	Dynamic analysis	✗	✗	✗	✗	✗	✗	✗	✓	✗	✗	✗	✓	✗	✗	✗	✗	✗	✗
Analysis	Static analysis	✗	✗	✗	✗	✗	✗	✓	✗	✗	✗	✗	✗	✗	✗	✗	✗	✓	✗
	Formal verification	✓	✗	✗	✓	✓	✗	✓	✗	✗	✓	✓	✓	✗	✗	✓	✓	✗	✓

**Table 3:** Summary of the installation of the security tools.

Tool	Ease of Installation	Usefulness	Stays Up to Date	Dependencies
Oyente	1/5	3/5	No	Python 2 (pip2), Z3 Prover, web3 (pip3), solc, Go-Ethereum
Remix-IDE	5/5	5/5	Yes	nodejs, npm
Solgraph	4/5	5/5	No	nodejs, npm, graphviz
MadMax	4/5	5/5	No	Python 3
Manticore	5/5	5/5	Yes	Python 3 (+3.6v), pip3, solc
SmartCheck	5/5	5/5	Yes	npm
Mythril	5/5	5/5	Yes	npm, Python 3, pip3
ContractLarva	3/3	3/3	Yes	Haskell (ghc)
SolMet	5/5	5/5	No	Java, maven, CSV reader
Vandal	4/4	4/4	No	Python 3
EthIR	1/5	3/5	Yes	Python 2 (pip2), Z3 Prover, web3 (pip3), solc, Go-Ethereum
MAIAN	1/5	1/5	No	solc, Z3, Python 3, web3
Erays	5/5	3/5	No	graphviz, Python
Rattle	5/5	5/5	No	graphviz, solc, Python 3
Osiris	2/5	5/5	No	Python 2 (pip2), Z3 Prover, web3 (pip3), solc, Go-Ethereum
Securify	5/5	5/5	No	soufflé, Java 8, solc
Slither	5/5	5/5	Yes	solc, Python 3
EtherTrust	1/5	2/5	No	Z3 Prover, Python, maven

## Abbreviations

The following abbreviations are used in this manuscript:

DAO	Decentralised Autonomous Organization
DLT	Distributed Ledger Technology
ETH	Ether (Ethereum cryptocurrency)
EVM	Ethereum Virtual Machine
P2P	Peer to peer
TEE	Trusted Execution Environment
